# Erector Spinae Plane Block in the Emergency Department for Upper Extremity: A Case Report

**DOI:** 10.5811/cpcem.2021.3.51803

**Published:** 2021-07-28

**Authors:** Daniel H. Lee, Marc L. Martel, Robert F. Reardon

**Affiliations:** Hennepin County Medical Center, Department of Emergency Medicine, Minneapolis, Minnesota

**Keywords:** erector spinae plane block, regional anesthesia, upper extremity, case report

## Abstract

**Introduction:**

The erector spinae plane block (ESPB) has been described as an effective analgesic modality in the emergency department (ED) for thoracic pain. It has not previously been described to treat ED patients with pain in the upper extremity.

**Case Report:**

We present a case of a 52-year-old female who presented to the ED with an acute exacerbation of her chronic radicular left arm pain originating after a fall she sustained one year prior. After a variety of analgesic modalities failed to control her pain, an ESPB was used to successfully treat her pain and facilitate discharge from the ED.

**Conclusion:**

A significant portion of patients who present to the ED have underlying chronic pain; however, opioids are a potentially dangerous and ineffective modality to treat chronic pain. In addition to avoiding opiates, the ESPB has the advantage of preserving motor function, thus avoiding the complications associated with brachial plexus blockade.

## INTRODUCTION

In the United States up to 20% of adults are estimated to be experiencing chronic pain at any given time, and up to 40% of emergency department (ED) patients have underlying chronic pain conditions.^1^ A variety of analgesic modalities are required to better aid these patients, as opioids are not indicated for treatment of chronic pain in the ED (excluding cancer patients).^2^ The potential risk of opioid misuse and abuse is increased in patients with chronic pain.^3^,^4^ The erector spinae plane block (ESPB) has been described to treat pain from acute conditions such as fractures, burns, herpes zoster, renal colic, and acute pancreatitis.^5^ The use of ESPB has the potential to be expanded to patients with upper extremity pain, as it has the distinct advantage over brachial plexus blockade of preserving motor function. We present a case report of a patient in the ED with chronic upper extremity pain who experienced significant improvement after undergoing an ESPB.

## CASE REPORT

A 52-year-old female with a history of fibromyalgia, left shoulder osteoarthritis, and chronic pain in her left arm, neck and back presented to the ED with an exacerbation of her chronic pain for two days. She had suffered a fall while in the shower one year prior to presentation and attributed her chronic neck, back, and radicular left arm pain to this injury. She had tried chiropractic manipulation, acupuncture, intraarticular glucocorticoid injections, physical therapy, and topical creams and patches, as well as a variety of over-the-counter medications. Despite these interventions, her pain persisted. Her vital signs on presentation to the ED were all within normal limits, and on physical examination her pain was rated as 10/10 on the left side including her lateral neck, back, circumferential upper arm, and lateral aspect of her elbow.

**Image 1:**
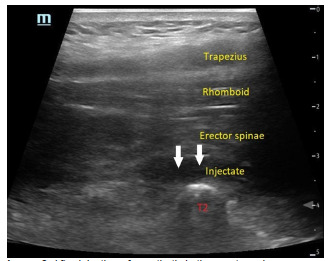
Sonographic anatomy prior to injection viewed with a linear probe in longitudinal orientation showing the erector spinae muscle overlying the transverse process of the second thoracic (T2) vertebrae. This is approximately 3 centimeters lateral to midline. White arrows indicate the surface of the transverse process where injection into the fascial plane is performed.

**Image 2:**
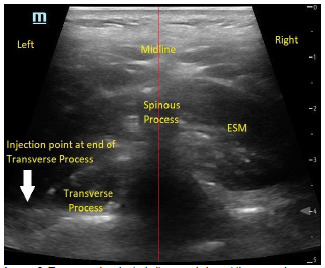
After injection of anesthetic in the erector spinae plane using an in-plane approach with the probe in parasagittal orientation. White arrows indicate the injectate seen as the anechoic stripe between the erector spinae muscle and the transverse process of the second thoracic (T2) vertebrae.

**Image 3:**
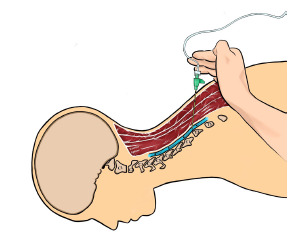
Transversely oriented ultrasound view at the second thoracic (T2) vertebrae showing the spinous process, which indicates midline on the patient’s back. The erector spinae muscle (ESM) is visualized directly lateral to either side of the spinous process. The left transverse process is also visible as the hyperechoic linear structure deep and adjacent to the spinous process. The injection point for the erector spinae plane block is marked with the white arrow at the edge of the transverse process.

She exhibited allodynia in these regions and had pain-limited range of motion at the shoulder and elbow. There were no areas of skin erythema, induration, or fluctuance. Plain radiographs of her shoulder and elbow revealed no acute findings. She had received 10 milligrams of oxycodone while waiting in the triage area, but this gave her minimal symptom relief, likely due to the severity of her pain. After explaining the risks and benefits to the patient, an ultrasound-guided ESPB was performed with 60 milliliters of 0.25% ropivacaine (Images 1–3).

**CPC-EM Capsule**

What do we already know about this clinical entity?

*Patients with chronic pain frequently present to the emergency department (ED). Chronic upper extremity pain can be treated with brachial plexus blockade, but this results in motor paralysis*.

What makes this presentation of disease reportable?

*The erector spinae plane block (ESPB) has not been previously used in the ED to treat upper extremity pain and may be an effective analgesic modality for these patients*.

What is the major learning point?

*The ESPB can treat chronic upper extremity pain without causing motor blockade. It is a safe procedure that can be performed by emergency physicians*.

How might this improve emergency medicine practice?

*The expanded indications for ESPB will allow emergency physicians to use regional anesthesia for chronic pain while avoiding unnecessary complications and opiates*.

The block was performed at the level of the second thoracic (T2) vertebrae with the patient in prone position and the ultrasound probe oriented parasagittally. An in-plane approach was used with the needle tip oriented cephalad. During the instillation of the local anesthetic, manual compression caudal to the site of injection was applied to influence spread of the injectate cephalad toward the vertebral levels where the patient was experiencing pain (Figure).

The procedure was performed without complication. After 30 minutes, the patient reported complete relief of her neck, shoulder, thorax, and back symptoms and had complete restoration of range of motion. She did not experience any motor blockade and had full strength in her extremity. She rated her pain at a 0/10 and expressed satisfaction at the quality of her pain control. Only minimal elbow pain persisted and after a period of monitoring in the ED, the patient was discharged home. On follow up, she reported that she had complete relief of pain for the next five days, after which her symptoms gradually returned at a more tolerable level.

## DISCUSSION

Regional anesthesia blocks can be an effective analgesic modality in patients with chronic pain. Patients with upper extremity pain are usually limited to brachial plexus blockade, which carries the risk of neural injury from inadvertent intraneural injection or needling.^6^ Furthermore, brachial plexus blocks affect both myelinated A motor fibers and unmyelinated C nociceptive fibers, causing patients to temporarily lose motor function in the affected limb.^7^ This does not allow for safe disposition of most patients from the ED. Brachial plexus blockade can also cause diaphragmatic paralysis, Horner’s syndrome, and central neural blockade, all of which are undesirable and potentially dangerous.^8^

The ESPB may provide a better alternative in these scenarios, where significant analgesia can be provided in the upper extremity without the disadvantage of blocking motor function and complications associated with brachial plexus blockade.^9^,^10^,^11^ The absence of motor blockade is thought to occur due to the low volume of anesthetic reaching the actual nerve roots and preferential blockade of nociceptive fibers.^10^ One study showed that computed tomography reconstruction of the ESPB performed at T2 demonstrated the spread of injectate superiorly into the cervical levels from the second through sixth cervical vertebrae (C2–C6), as well as anteriorly past the levator scapulae muscle.^11^ Cadaveric studies where the ESPB is performed using methylene blue have also consistently demonstrated extensive spread of injectate to the ventral and dorsal rami across multiple vertebral levels.^12^ This explains the ability of an ESPB performed at the level of T2 to spread cephalad through fascial planes and block cervical nerves.

For rib fractures, the ESPB is typically performed at the T3–T5 level with the injection needle oriented in a cephalad to caudal direction to facilitate downward spread of the local anesthetic. In this case, in order to have the injectate spread cephalad, the needle was directed from a caudal to cephalad direction at T2. This process allowed more cephalad spread of injectate toward the cervical spine in an attempt to alleviate the patient’s chronic upper extremity and neck pain without motor blockade.

Ropivacaine is expected to have a duration of 12–24 hours, which can be extended up to six days if administered with appropriate doses of dexamethasone and epinephrine.^13^ The patient in this case received 0.25% ropivacaine without additives but experienced several days of complete pain relief. This suggests that the ESPB interrupted a pain cycle. as the anesthetic effect alone does not explain the duration of her analgesia.

In contrast to trigger point injections that use needling to disrupt hyperirritable intramuscular nodules in patients with myofascial pain syndrome, the ESPB is thought to anesthetize peripheral nerves and can be applied to any patient with acute or chronic pain.^14^ The mechanism of trigger point injections has been questioned for decades, and it is postulated that some trigger point injections provide analgesia by inadvertently blocking peripheral nerves when local anesthetic is injected.^15^ Thus, the mechanisms of trigger point injections and the ESPB may overlap to some degree; however, no comparison studies between the two are available.

The exact mechanism for preservation of motor function is not understood. Only case reports have been published about this method. It is possible that a large-scale study would uncover patients who inadvertently received motor blockade with this technique. Although needle direction and manual compression were used in this case to influence anesthetic spread, it is unknown whether these maneuvers improve efficacy of the ESPB, and this technique is currently without high-quality evidence.

## CONCLUSION

The erector spinae pain block may be a safe and effective modality to treat chronic upper extremity pain in the ED, without significant blockade of motor function. Performing the ESPB in the high thoracic region, aiming the needle cephalad and applying compression below the site of injection, may help the anesthetic spread upward into the region of the cervical nerve roots. Research is needed to determine the utility and efficacy of this new technique.

## Figures and Tables

**Figure:**
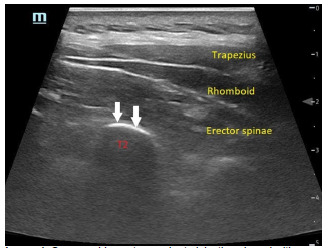
Depiction of manual compression during injection of anesthetic as well as the cephalad orientation of the needle. Black arrows indicate the direction of injectate flow (Illustration by Elizabeth Lee).
